# “It was Very Liberating”. Dialogic Literary Gatherings Supporting Mental Health Literacy

**DOI:** 10.1007/s10597-022-01071-0

**Published:** 2022-12-27

**Authors:** Harkaitz Zubiri-Esnaola, Sandra Racionero-Plaza, Aitana Fernández-Villardón, Sara Carbonell

**Affiliations:** 1 Department of Language and Literature Didactics, University of the Basque Country UPV/EHU, San Sebastian, Spain; 2 Department of Sociology, University of Barcelona, Barcelona, Spain; 3 Faculty of Education & Sports, University of Deusto, Bilbao, Spain; 4 Faculty of Education, University of Girona, Girona, Spain

**Keywords:** Mental health, Health literacy, Mental disorders, Mental health literacy, Dialogic literary gatherings, Successful educational action

## Abstract

Mental health is being reframed as a fundamental right for all people, and mental health literacy is a tool that can enable patients to gain the knowledge, personal skills, and confidence to take action to improve their mental health, and their lives overall. This exploratory study analysed the power of dialogic literary gatherings (DLGs) to foster it in a group of patients with mental health disorders who gathered for 1 h once a week to share their readings of literature masterpieces. During the year-long study, a total of 140 patients participated in the DLGs in groups of 12 to 15 people. Results suggest that DLGs promoted the development of the participants’ mental health literacy and produced gains in emotional and social wellbeing by strengthening reading, speaking, and listening skills, fostering supportive relations, contributing to overcoming stigma, and enhancing agency. The transferability of DLGs to mental health care is discussed.

## Introduction

The approach to mental health is currently undergoing a profound transformation due to the convergence of evidence from numerous scientific areas, the WHO’s (2013) Comprehensive Health Action Plan, several international agreements adopted in recent years, and the progressively more pronounced consensus of some stakeholders on how to address the challenge (Patel et al., 2018). The emerging approach implies (1) reframing mental health as a fundamental human right for all people, (2) considering that the mental health of each individual is the result of the interaction between social, environmental, genetic and psychological processes, and (3) expanding the current clinically defined perspective to a multi-dimensional approach to mental health including social interventions along with psychological and pharmacological treatments (Kleinman et al., 2003; WHO, 2013; Patel et al., 2018).

This emerging approach to mental health is framed in a rights-based perspective in which human rights, equity and scientific evidence are the fundamental principles (Barry et al., 2013; Asher et al., 2017; Patel et al., 2018; Purgato et al., 2018). In 2015 the United Nations General Assembly approved the sustainable development goals (SDGs) (UN, 2015). Goal 3 of the SDGs points to “ensure healthy lives and promote well-being for all at all ages”, and target 3.4 focuses on promoting “mental health and well-being”. The SDGs go beyond the idea that there is no health without mental health and emphasise that there is no sustainable development without mental health. Mental health is a global public good that concerns not only those biomedically defined as suffering a mental disorder but as a universal human attribute which is an indispensable part of overall health, and therefore is essential for all kinds of people of all ages in all countries (Patel et al., 2018).

Increasing investment in mental health care is necessary but not sufficient to meet the challenge of an equitable perspective that takes into account the right to the mental health of all persons without exception, since there are other gaps that must also be addressed: a care gap (Kohn et al., 2004) and a quality gap (Patel et al., 2018). In fact, there are countries (for example, Australia, Canada, England, and the USA) where there has been a significant increase in the provision of treatment, yet this has not led to a decline in the prevalence of mood and anxiety disorders and symptoms, even though the risk factors have not increased (Jorm et al., 2017).

It is therefore necessary to identify actions that can contribute to overcoming the attention gap and the quality gap. The present study contributes in that direction by means of exploring the ability of a particular intervention, the dialogic literary gatherings (DLGs), to be one of those effective actions. Specifically, the study we report here, analyses the impact of DLG on mental health literacy. Research has shown that promoting health literacy is beneficial to mental health. In addition, scientific literature indicates that health literacy implies strengthening reading, speaking and listening skills (Berkman et al., 2011; Nouri & Rudd, 2015; Rudd, 2019), fostering supportive relations (McCormack et al., 2017; Samerski, 2019; Rudd, 2019), contributing to overcome stigma (Jorm, 2012; Corrigan et al., 2014), and enhancing agency (Jorm, 2012; Corrigan et al., 2014), among other aspects. Research has also demonstrated that those four aspects are also fostered by DLGs (Flecha, 2015). Thus, this study analyses the impact of DLGs on the promotion of these fundamental aspects of Mental Health Literacy.

The following first sub-section reviews in more detail how these four aspects (1. reading, speaking, and listening skills; 2. supportive relations; 3. stigma; and 4. agency) relate to mental health literacy, while the second sub-section discusses the characteristics of DLGs as well as the improvements they generate and in which contexts they have been analysed so far.

### Mental Health Literacy

#### Reading, Speaking, and Listening Skills

Health literacy has been defined as a set of skills to locate, understand, interpret, use and express information adequately regarding personal health (Berkman et al., 2011). There is a clear relationship between general literacy and health literacy (Rudd, 2007), and reading skills as well as oral (speaking) and aural (listening) literacy skills considerably influence health outcomes (Federman, et al., 2009; Macek et al., 2010; Sorensen et al., 2012; Nouri & Rudd, 2015; Rudd, 2019). Poorer literacy is not only related to poorer access to information to manage one’s own health but also to lower participation in activities that help prevent disease (Rudd, 2019).

#### Supportive Relations

Most definitions consider health literacy as an individual cognitive ability (Samerski, 2019). However, there are studies that question this perspective and conceptualize health literacy as a multidimensional social practice that is co-produced in social relationships (Samerski, 2019). Research that assumes that interactions are the basis of health literacy shows that physical, social and normative contexts can facilitate or hinder communicative practices, and therefore changes in communicative practices can facilitate health literacy and consequently influence health outcomes (Rudd, 2019). A health literacy social ecological model takes into account that the individual is influenced by interwoven contextual elements, and, therefore, the social-ecological model targets the individual as well as multiple levels of influence such as interpersonal and community in order to create supportive environments, so that these interactions generate synergies between levels, enhance patient involvement and lead to more sustainable changes (McCormack et al., 2017). In fact, research has shown that supportive relations significantly influence the level of satisfaction with one’s life Harvard Study on Adult Development (1938-2022), health in general (Holt-Lunstad et al., 2010), and mental health in particular (Lakey & Orehek, 2011).

#### Stigma

Overcoming stigma, discrimination, social exclusion, and human rights abuses is one of the major challenges for the emerging approach to mental health (WHO, 2019). In fact, people with mental health conditions are considerably stigmatised (Corrigan et al., 2014; Antonelli-Ponti et al., 2021; Fung et al., 2021). A considerable amount of people prefer to establish social distance from people labelled as having mental disorders (Angermeyer et al., 2009). Furthermore, people with mental illness often report discrimination in health care (Thornicroft et al., 2007).

Mental health literacy can be useful in helping to overcome stigmatisation (Jorm, 2012). Effective interventions can increase health literacy (Pinto-Folz et al., 2011) and, as knowledge about how stigma works increases, its negative impact moderates (Corrigan et al., 2014). However, professional contact and treatment do not necessarily lead to promoting mental health literacy (Goldney et al., 2002) and not all types of mental health literacy decrease stigma (Schomerus et al., 2012).

#### Agency

A wide definition of health literacy focuses on increasing the autonomy and personal empowerment of patients, thus contributing to more effectively bridging the health gap that exists between people in different socio-economic positions (Nutbeam, 2000). The WHO (1998) definition of health literacy is also within the framework of empowerment, as it refers to acquiring a level of knowledge, personal skills and confidence to take action to improve personal health and is itself dependent upon more general levels of literacy.

The term mental health literacy was coined in 1997 and defined as “knowledge and beliefs about mental disorders which aid their recognition, management or prevention” (Jorm et al., 1997). It is an evolving term that should be interpreted in the context of health literacy and its components (Kutcher et al., 2015a), and it has also been expanded so that it now includes “the range of cognitive and social skills and capacities that support mental health promotion” (CAMIMH, 2007, p. 36). Now it also involves aspects such as fostering supportive relations (Jorm, 2012, p., 6) and decreasing stigma (Corrigan et al., 2014; Kutcher et al, 2015b), and it is in line with the interpretation of health literacy as empowerment (Kutcher et al., 2015b). Thus it “involves spreading the expertise for dealing with mental disorders across the whole community” (Jorm, 2012, p. 10) and so enhancing agency (Jorm, 2012; Corrigan et al., 2014; Kutcher et al., 2015b).

The promotion of health literacy is closely related to the necessity of tailoring appropriately messages to the needs and preferences of the targeted people (Kelly et al., 2007; Kermode et al., 2009). Whether patients and families can make sense of the initiatives that lead to recovery is a major challenge for these actions to be truly successful (Patel et al., 2018). In fact, many people affected by mental disorders do not want to receive treatment, due to poor awareness of the illness (Belvederi & Amore, 2019), and this contributes to an increase in the number of people in need of care who do not receive it (Degenhardt et al., 2017; Thornicroft et al., 2017; Alonso et al., 2018).

It is necessary to adjust the actions to the needs of the people concerned so that they do not reject the actions offered, and stay involved and actively engaged being the protagonists of their own recovery process (Kelly et al., 2007; Kermode et al., 2009; Patel et al., 2018). When facing health challenges, depending on the circumstances, sometimes the patients involved do not use their personal competences, do not make sense of what they are doing and do not engage (Patel et al., 2018). In order to overcome these obstacles, it is essential that the actions taken to develop mental health literacy connect directly with their experiences and views (Patel et al., 2018) in order to foster personal acceptance of the illness suffered, which is linked to good prognosis (Kao & Liu, 2010).

### Exploring DLGs as an Effective Mental Health Literacy Intervention

Psychosocial interventions, which include talking therapies and social interventions, can be very successful in creating social circumstances conducive to patient recovery (Patel et al., 2018). Encouraging collaborative interpersonal contact is the most effective way to help increase the acceptability of treatment and overcome stigma and discrimination (Patel et al., 2018) as well as to strengthen social bonds by building relationships with other people including people with a mental health condition, and thus increase the chances of weaving a social support network that will make it easier for the patients to continue with the activities that promote recovery and so make changes in their lives (WHO, 2019).

Dialogical literary gatherings (DLGs) (Flecha, 2015; Soler-Gallart, 2017), which is defined as a Successful Educational Action by the European Commission (INCLUD-ED, 2009), may respond to these challenges very effectively. In DLGs interpersonal egalitarian dialogue about universal classical literature is the basis for empowering the participants to develop views and skills that contribute to transforming their lives (Flecha, 2000).

DLGs consist of choosing a book from among the classics of world literature, agreeing among participants which chapters to read, reading what has been agreed upon and underlining the sentences that are considered interesting, and then getting together to read and comment on the paragraphs that have been underlined (Fernández-Villardón et al., 2021). The role of the moderator is not to lecture on the analysis of the book but to ensure that mutual respect and interest prevail and that everyone feels comfortable and legitimated to participate and thus enrich the conversation (García et al., 2017b). DLGs offer the possibility of reading and commenting on a book that contains timeless topics such as love, war, social relations and everyday life challenges that concern all human beings regardless of their particular circumstances (Ruiz-Eugenio et al., 2020).

Reading and commenting on a classic book in DLGs lead to experiencing works of art that, contrary to what is often claimed, are accessible to everyone, not just to those belonging to the so-called *high culture* (Soler-Gallart, 2017; Torras-Gómez et al., 2021; Lopez de Aguileta, 2021). Research has shown that DLG promotes language and communication skills (Flecha et al., 2013; García et al., 2017a; López de Aguileta, 2019; Santiago-Garabieta et al., 2022) as well as relationships of friendship and respect that are felt to be enriching (Flecha, 2015), a sense of community (García et al., 2017a), solidarity (García-Carrión, 2015), prosocial behaviour (Villardón-Gallego et al., 2018) and improvements in terms of emotional well-being, self-concept and self-esteem of the participants (Flecha et al., 2013; García et al., 2017a), so that it helps to overcome stereotypes (García et al., 2017a) and especially people with more difficult life trajectories live a truly inclusive experience in DLGs (Flecha, 2015).

DLGs have proven to be successful in diverse geographical and cultural contexts (Flecha et al., 2013; Alvarez et al., 2018) and has the capacity to foster the social inclusion of vulnerable groups (Flecha, 2015). Initially, DLGs were developed in adult education, and later it was successfully transferred to Infant, Primary and Secondary Education (Sanchez, 1999; Flecha & Soler, 2013; Flecha, 2015; Villardón-Gallego et al., 2018). Subsequently, DLGs have been developed in many other contexts with positive results: prisons (Flecha et al., 2013; Alvarez et al., 2018), women of immigrant origin (García et al., 2017a), women over 60 (García et al., 2017b), homeless people (Racionero-Plaza, 2015), and children in care (García et al., 2017a).

## Methods

### Purpose

The new approach to mental health demands identifying actions that can address the treatment and quality gaps from a perspective based on human rights, equity, and scientific evidence because the emerging approach reframes mental health as a fundamental right for all people, and addressing this challenge requires a multi-dimensional approach that includes both psychological and pharmacological treatments along with social interventions. The study reported in this manuscript seeks to respond to that need by investigating whether the DLGs implemented with patients with mental disorders are effective as such kinds of interventions. Specifically, the investigation addressed the following research question: Does DLG contribute to the development of mental health literacy in terms of improving reading, speaking, and listening skills, supportive relations, agency and overcoming stigmatisation in patients suffering a mental health condition? If so, how?

### Research Approach

The research was conducted using the communicative methodology of research (Gómez et al., 2011, which has been recommended by the European Commission for its proven effectiveness in raising the social impact of research. In this methodological approach, knowledge of the reality studied is co-created through researchers’ egalitarian dialogue with research participants (Sordé et al., 2020). The aim of the communicative methodology is to identify solutions to problems identified by citizens, so that the research can provide citizens with resources that serve to improve the lives of those involved in the area under investigation (Munté-Pascual et al., 2022). The communicative methodology of research has been successfully applied in the study of problems affecting vulnerable groups (Gómez et al., 2011) as well as in all the research examining the impacts produced by DLGs (García-Carrión et al., 2020; López de Aguileta et al., 2020; Ruiz-Eugenio et al., 2020).

### Implementation

The research was conducted in a public hospital located in a Spanish city, involving users of two units: the Sub-acute Unit of the Day Hospital and the Psychiatric Dependency Unit. It was a patient who proposed to the hospital’s multi-professional staff to apply DLGs. Afterwards, the staff requested training from an Adult Education Centre near the Hospital which had been implementing DLGs since the 1980s. The multi-professional team of the Hospital was trained by a member of the Adult Education Centre and a researcher from the Community of Researchers on Excellence for All, the research Centre that has scientifically investigated DLGs for decades. After the training, DLGs were initiated in the hospital. DLG sessions were moderated by volunteers, one woman who was in charge as a volunteer of a DLG in an Adult Education Centre, and one man who was a teacher in an Adult Education Centre. The investigation reported here was carried out in the second year of implementation of DLGs, from fall 2016 to spring 2017.

There were about 12–15 participants in each DLG session, and a total of about 140 participants in one year. It was an open group, with people joining and people leaving throughout the year. All patients who participated had severe mental disorders, but each participant was at a different point in the recovery process. Additionally, 2 volunteers from the field of social intervention and 2 nurses participated in the sessions. The age of the participants ranged from 18 to 60 years. Both men and women participated. The hospital served patients with diverse socio-economic backgrounds: about 60% of the patients were characterised by low socio-economic status, many of them with severe social problems and very low levels of literacy, while about 40% of the patients came from medium and high social backgrounds, including people with university degrees.

The sessions took 1 h per week. The books were chosen by the participants from UNESCO’s list of masterpieces of the world heritage. The participants read, among others, Franz Kafka’s Metamorphosis, William Shakespeare’s Hamlet, Pedro Calderón de la Barca’s Life is a Dream, and Jules Verne’s Journey to the Centre of the Earth. The pages to be read for the next session were agreed upon by the participants.

### Data Collection

The study followed the design of a qualitative case study. The patient who proposed to do DLGs in the hospital and a nurse engaged in the activity were interviewed. These interviews were audio and video recorded. The script of the interviews was focused on capturing what was done in the DLGs and the impact it had on the patients and the professionals involved. In addition to this, 4 DLG sessions (4 h in total) and 1 communicative discussion group about DLGs with frequent participants in DLGs were audio recorded.

### Data Analysis

The interviews and recordings of DLG sessions were transcribed and clustered into topics, which were decided based both on the scientific literature about mental health literacy and DLGs. Following this deductive approach, four categories were defined: (1) reading, speaking, and listening skills, (2) supportive relations, (3) overcoming stigmatisation, and (4) agency. Researchers discussed data analysis and reached agreements whenever different interpretations of the data arose.

### Ethics Statement

This research meets the research ethics criteria set out in the European Commission’s (2013) ethics review procedure. The design of the research was approved by the multi-professional staff of the hospital. The objectives and characteristics of the research were explained to all participants. Both professional staff and patients participating in the DLG sessions and in the interviews gave their written informed consent. The written consent document included the goals of the research and a clear explanation that participating in the research was voluntary and could be terminated at any time. It also guaranteed anonymity and confidentiality. The study was also revised and approved by the Ethics Committee of the Community of Researchers on Excellence for All (CREA) under reference number 20,210,422.

The authors report there are no known conflicts of interest. All authors certify responsibility.

## Results

### Strengthening of Reading, Speaking and Listening Skills

The patients who participated in DLGs finished reading all the works that were chosen, and they completed a one-hour gathering per week. Reading was a necessary condition for participation in DLGs. Everyone who participated in the discussion had read the chapters they had agreed upon and underlined the phrases that had struck them as being noteworthy. However, reading was not merely an individual task, but the key aspect highlighted by the participants was sharing the reading with others. Reading to share in DLGs was very encouraging to focus on a task and to be persistent. In the words of one participant: “It helps to encourage us to read or, in the case of poor concentration, helps to force us to make the effort to read because of the commitment it entails”.

Furthermore, dialogues in the DLGs promoted reading comprehension and encouraged better recall of reading cues. The nurse interviewed explained it this way: “The patients say that (…) on Monday we’ll reread it again and everyone’s opinions make me think in a way that the text stays with me, that I can retain it, that I can memorise something of what I’ve read.” One participant also said that “sharing ideas facilitates understanding”.

According to the participants who were interviewed, the dialogical nature of the activity is what encourages the development of communication skills such as the capacity to sit, concentrate and be attentive to the task, and the ability to respect speaking turns, all of which are essential for developing the ability to communicate dialogically.

Interviewed nurse: “The experience with all these books has been very interesting, it has been amazing. (…) It is a pleasant surprise to see that you can do these literary gatherings. (…) They can follow these literary dialogical gatherings because the gatherings are dialogical, and that is the key. (...) For me as a professional it was unthinkable. If you tell me years ago that I would be part of a literary conversation with the patients admitted in sub-acute, I would have said no, that it was not possible. Well, it’s possible. (…) Sometimes, the patient with a mental disorder can be dispersed, it is difficult for them to concentrate, and it is difficult for them to be still. So what also surprised us in the first sessions of DLGs was that the patient could be seated. We were all sitting. This environment allows each patient to be seated as well. (...) Very rarely have patients had to get up and leave. (…) So [DLG] helps them to be attentive to something concrete, to be able to listen, to keep their attention, to keep their turn to speak. (…) That’s why keeping your turn to speak helps us regain social skills we’ve lost that will be useful in our lives when we’re discharged.”

The patient interviewed corroborated that the DLGs significantly encouraged participants to concentrate on the task: “In the [DLGs] I immersed myself in what we were reading.”

Moreover, the contributions of some participants triggered and shaped others’ contributions, and the participants always expressed full respect for each other’s interpretations. For example, one of the participants stated the following while speaking in a DLG session about Jules Verne’s *Journey to the Centre of the Earth*: “In DLGs, I have seen that you can express yourself with ease and without prejudice.” Another patient interviewed shared the same perspective: “In the dialogue that is established, which is a dialogue that one person has never stepped on another, turns are respected, every contribution is welcome, no one is criticised for having a different idea, and gives you a lot of freedom as a person, and also, you acquire a lot of cultural baggage.” This informs of a respectful atmosphere, based on the freedom to speak, which is a remarkable feature for DLGs being a space where communication skills can be developed.

### Fostering Supportive Relations

DLG sessions provided opportunities to discuss social relations in depth. DLG conversations linked comments on the psychology of the characters and events in the story with the personal experiences and opinions of the patients. In this regard, many were the topics discussed in DLGs: friendship, hope, suicide, things that change inexplicably, and the influence that our immediate environment has on people. Underlying all these issues, it was clear the importance the participants of DLGs attached to the support that the people around them could provide, so that changes at the personal level could become a reality. Specifically, the analysis of the data collected suggested that in DLGs the participants reinforced the idea that individual transformations necessarily require a supportive and safe environment, and when it is not in place, making improvements is much harder. This reflection was particularly prominent in the discussion of Franz Kafka’s *Metamorphosis*. During those sessions, the conversations in DLGs clearly linked the importance of communication and mutual care for well-being. The patients frequently highlighted the importance of weaving networks of relationships that make it possible to face the difficulties that arise in life. The participants described the family in *Metamorphosis* as having problems that should be overcome for mental health, such as isolation and lack of communication. The dialogues among patients in the DLGs also suggested mutual care and love as goals to be achieved to overcome those family problems:

Participant 1: “The concern she is having with her brother (...) She is the only one who is doing something for him. (...) Why do parents also think their daughter is useless? I don’t know, and I also think that Gregor isn’t very well considered either, even when he was normal. (...) I don’t like this relationship in the family, it’s not healthy, that’s what amazed me. (…)”

Participant 2: “Well, it is another example of a lack of communication with others and oneself. (...) She certainly doesn’t see herself with good eyes because she is no longer the good sister she wanted to be, does she?” (…)

Participant 3: “I have also underlined those phrases that [another participant] named, (…) because I was thinking about it, in total desperation. I thought that it was a family that was isolated from everyone and that has no help from anyone. And there are alternatives. (...) They are stuck, in total desperation. If misfortune happens to us now, we have support. If it is due to illness, there is help, there are nurses, doctors…” (…)

The following is another example of how one of the participants in the DLGs identified her personal situation with the situation of partial isolation experienced by one of the characters in *Metamorphosis*. The patient chose this excerpt from the book: “Climbing on the chair, leaning against the window, leaning through it, no doubt as memories of how free she had always felt when she had been leaning against it”. Once she read it aloud in the group, she followed with her interpretation, explaining that the text made her think a lot about herself because “we partly have a regime of freedom here, but we also have a regime of confinement.” She was referring to the confinement in which they lived due to the mental disorder.

Unlike in *Metamorphosis*, when analysing Jules Verne’s *Journey to the Centre of the Earth*, the participants in DLGs identified beneficial features in the characters of the novel. For example, one of the participants said that she “was struck by the fact that they helped each other and there was a companionship between them. If there was no friendship, they would have died”. Another participant highlighted the importance of “trusting a person with closed eyes” and valued one of the characters because “Hans is a person who is attentive to others”.

Overall, the data analysed indicated that participating in DLGs led to generating, at least for some patients, a sense of closeness, trust and connection to others, and to building a sense of community. One participant put this as “sharing reading makes us accomplices”, which comforted patients feeling that they were moving away from the isolation that mental illness had brought them. A patient explained this very clearly:

Interviewed patient: “ [DLGs] helped me a lot to get through those days when I was in a very bad situation. (...) They gave me a lot of support. (...) and when I heard other people’s expressions, and comments, my head was really moving away from daily problems. (…) I believe that DLGs can serve to disconnect them from their inner world. When you are suffering an illness, it is you with your inner world, it is you with your thoughts facing your inner world. And in this way, you show yourself to the world. It’s a way to open up to others, and to know that you’re not just a number or a patient, but that you’re a person, that you have your own criteria, that there are people who listen to you, and that’s very thankful, that there’s someone who listens to your opinions. (…) It was very liberating, and you also got involved with the group, and you could also give your opinions. (...) That contact with the world outside of me helps me a lot. (…) Having that moment with people you already knew and with whom you already had a friendship, because we were a family, that helped me a lot to grow as a person and to face my illness.”

DLGs offered supportive relationships that contributed to the recovery and were not substitutes for professional help, but rather were in conjunction with the participation of professionals and as a complement to add to and complementary to professional help.

### Overcoming Stigmatisation

When participants in DLGs shared their opinions about the readings, the comments on the contents of the book mingled with personal experiences, feelings, and concerns. Analysing the characters’ psychology and the characteristics of the events led to co-constructing interpretations of common problems such as stigmatisation, which was a recurring theme in the meetings. When the patients discussed this, they re-constructed the interpretation of their trajectories of exclusion and stigma.

The relationship between the analysis of the fictional characters and situations and the analysis of the participants’ lives was explicitly put into words sometimes, and when the connections were not made openly, they were easily felt in the conversation. For instance, in the excerpt below, when commenting on Franz Kafka’s *Metamorphosis*, the conversation focused on the problem of being stigmatised, which was identified as a central challenge for both the characters in the book and the patients participating in the DLGs.

Participant 1: “despite his disgusting present form(…) [Gregor] is a family member who could not be treated as an enemy, (…) someone to have locked up, not to be seen. (…) You seek help because sometimes there are situations that I think we cannot bear as people, right? We need help, because if not, then we give up. (…) There must be love between them, that’s what I think would save them.” (…).

Participant 3: “I think it’s a way for the writer to explain in one way how he feels and, in another way, how the family has despised him and then gives the example of the bug.” (…).

Participant 4: “Well, my relationship with my family is bad, so I’m starting from that base. (…) I was thinking about the bug, if you imagined it, if it was a freak and that’s why they marginalised it or something like that, that’s what I was imagining.”

Participant 5: “That’s what we’re all in.”

[Everyone laughs] (...)

Participant 6: “You haven’t met that sometimes it’s said: I look like a freak. (…)You feel outside the group. (…)

The results also indicate that a unique trait of DLGs which is the reading of works of the classical literature was perceived by the participants as contributing to overcoming their stigmatization as mental health patients. The literary classics, identified as socially prestigious and valuable, were seen by patients as a tool to reverse stigmatisation in practice. Indeed, the stigmatisation perceived by the participants in DLGs was not only based on their mental health condition but also on their socio-cultural position and academic trajectory. Thus, for instance, when one of the DLG sessions was about to finish, after the participants developed deep and moving reflections about Franz Kafka’s *Metamorphosis*, they concluded that prejudices do not hold up against the reality that occurs in every DLG session: those patients perceived that they were not supposed to read, have fun and take advantage of classics, but just as the conversations in the gatherings developed, they proved to themselves in practice that the prejudices were not true and could thus open up new possibilities for improvement in their life paths. Three patients shared this in the following way:

Participant 1: I liked the book very much, and I don’t know, here we are thinking about things you say… well, there are a lot of things that come out… if we read the book, we imagine things it goes far beyond what we simply read. That’s why maybe they [she refers to academic people] say they can only understand them….

Participant 2: Those people with university studies.

Participant 3: Well, that is how the lack of knowledge of others serves to prejudge some people.

### Enhancing Agency

The concern to develop the agency necessary to enable change was an issue that went through all conversations in DLGs. For example, in the excerpt below, a nurse, one of the participants in DLGs, emphasized the importance of the topic of destiny in DLGs about Pedro Calderón de la Barca’s *Life is a Dream*: “[In *Life is a Dream*] the subject of destiny also [came up], whether we are destined for something or how it conditions our life, how it conditions what happens to us, and what we can do, because much came out of what I can do so that we are not destined for a destiny. So that I can participate in my destiny. (…) It is not by destiny, which can be influenced by many things, it is by what I do.” The following is another example of how a similar idea appeared in the DLG on *Journey to the Centre of the Earth* when one of the participants said during a DLG that it is very important “to know that one can try everything in life with effort and tenacity” and “patience is the best travel company”. Along with the idea of striving for personal change, DLG dialogues also stressed the need to stick to objectives that would help in life, developing a meaning-making process among participants. For instance, when speaking about *Journey to the Centre of the Earth*, one of the participants emphasised that “you can’t miss the north. One must always be focused on something in order not to lose the north in life”.

Evidence collected also suggests that participants in DLGs did not understand building individual agency as merely an individual process. This was reflected in the dialogues that took place in DLGs. For example, when sharing their readings on *Journey to the Centre of the Earth*, one of the participants in DLGs said about one of the characters in the book that “being a supporting character, he is one of the important people on this trip” because “thanks to Hans they give credibility to the trip”. Likewise, when reflecting on DLG itself, the same idea appears for example when the nurse interviewed suggests that DLG sessions served to make a long-term type of agency prevail because having a supportive interaction between patients in different stages of their trajectory made it easier to create a long-term perspective of the individual agency. This occurred by making visible pertinent and useful personal references that may be used by other patients for contributing to transforming their lives:

Nurse: “[DLG] is part of the recovery as people, of the recovery of our capabilities. At the level of the patient’s recovery (…) I think it helps. (…) The group helps, because it’s not the same that a professional tells you: Well, you have to give time to time, little by little you will feel better... This helps. But another patient who is already on another itinerary telling it to you is a different thing. If after being admitted, the patient has started going to the day hospital, because this could be the case, the patient [who is entered to the sub-acute unit] sees that there is a whole process, and the one who says it is not a professional, it is another user who has already gone through the process that [another participant in the discussion] is going through. It helps the patient a lot to see that there is an evolution. [Sometimes they say] I’m fine, I must go... Then it helps.”

In addition, the data analysed suggests that DLGs can empower agency because DLGs provide opportunities to reinforce self-esteem when participants express and argue their ideas to others who may not always think the same way but with whom disagreements can be feasibly addressed. This is appreciated when, for example, the patient interviewed explains the following: “[DLGss] are an open discussion. (…) Having to expose your ideas or what you think about a certain aspect of the book, and trying to defend them, so to speak, that gives you a lot of self-esteem.”

## Discussion

Mental health is a fundamental human right for which mental health literacy is an important component. The present study provides evidence of a particular case suggesting that implementing DLGs in a Sub-acute Mental Health Day Hospital and in a Psychiatric Dependency Unit strengthened reading, speaking, and listening skills, fostered supportive relations, contributed to overcoming stigmatisation, and enhanced human agency. These gains are fundamental pillars of mental health literacy as a component of a wider definition of mental health and health literacy (Jorm et al., 1997; WHO, 1998; Nutbeam, 2000; CAMIMH, 2007, p. 36; Federman et al., 2009; Macek et al., 2010; Berkman et al., 2011; Flecha et al., 2011; Jorm, 2012; Sorensen et al., 2012; Corrigan et al., 2014; Nouri & Rudd, 2015; Kutcher et al., 2015a, 2015b; McCormack et al., 2017; Rudd, 2019; Samerski, 2019). Therefore, the improvement of these aspects means fostering mental health literacy for these patients.

Our study indicated that these improvements were related to engaging in dialogues in which episodes of works of universal classical literature awaken own experience and led to an awareness of one’s psychological challenges, a key aspect of rehabilitation (Chan et al., 2014). This occurring in an environment of respectful and supportive social relations promotes identification processes between participants and, thus, a greater understanding of the illness. These features of DLGs as enacted in the case studied align with the mental health literacy objective of empowering the community to act to benefit people with mental illness (Jorm, 2012). For the participant patients, DLGs supported skills that help connect with the community by practising enriching social relationships in which each patient can help others, and, at the same time, each patient can receive the support of the rest of the participants in DLGs for her or his emotional wellbeing.

Prior research had already shown that DLGs are effective in diverse settings, such as schools and prisons, and in diverse geographical locations and socio-economic environments (Sanchez, 1999; Flecha et al., 2013; Flecha & Soler, 2013; Flecha, 2015; Racionero-Plaza, 2015; Alvarez et al., 2018; López de Aguileta, 2021). The present study is a pioneer in contributing evidence from a particular case that DLGs can also be effective in mental health for supporting mental health literacy. This finding is of interest in the field of mental health for various reasons. One is to support implications in treatment and adherence to it, as research has shown that many affected people do not want to receive treatment (Degenhardt et al., 2017; Thornicroft et al., 2017; Alonso et al., 2018). Tailoring actions to the needs and preferences of patients (Kelly et al., 2007; Kermode et al., 2009) is a particular way that has been recommended to reverse that outcome. DLGs can be a promising intervention in that regard, as by definition DLGs are always adapted to the circumstances of the patients involved, as patients themselves establish connections between the content in the readings and their own experiences, feelings, and thoughts, often revisiting their own life trajectories, experiences with the mental disorder and future, impelled by the content of the books. Previous studies had already revealed that sharing experiences of mental health illness helped to improve connectivity with others and facilitate support-seeking (Lindstrom et al., 2021).

In addition, the improvements promoted by DLGs in the case analysed are in line with the emerging approach to mental health as a fundamental human right (Patel et al., 2018) and with the broader and more ambitious definition of mental health literacy (WHO, 1998; Nutbeam, 2000). This new approach to mental health, which emphasises the importance of equity, approaches mental health as a process involving social, environmental, genetic and psychological elements, and approaches mental health from a multimodal perspective that includes actions that complement the biomedical approach, engages professionals from various fields, and promotes the active participation of patients in their own recovery (Kleinman et al., 2003; Barry et al., 2013; WHO, 2013; Asher et al., 2017; Patel et al., 2018; Purgato et al., 2018; ). Mental health literacy is a tool to make the right to mental health a reality by enabling patients to gain the knowledge, personal skills, and confidence to take action to improve their mental health, and their lives overall (Nouri & Rudd, 2015; McCormack et al., 2017; Samerski, 2019; Rudd, 2019).

Our study provides data that points to the ability of DLGs to support mental health literacy through the effective development of key components of mental health literacy, such as reading, speaking and listening skills, supportive relations, agency, and the overcoming of stigmatisation, and does so by creating a context in which humanity’s best literary works provide the basis for dialogue that fosters enriching interpersonal contact and the sense of being part of a supportive community, so that patients are empowered in their agency to contribute to transforming their own reality. In this regard, DLGs could be tackling the care gap (Kohn et al., 2004) and the quality gap (Patel et al., 2018) that research has identified as major challenges regarding mental health.

This is a qualitative case study; therefore, our findings cannot be generalised; both with respect to other mental health centres and other profiles of people suffering from mental disorders. Our results only apply to the groups of patients analysed and to the specific health settings investigated. Also, in qualitative research, the gains reported by the participants in the interviews and focus groups are grounded in their perceptions. Further research could include the examination of the gains identified in our study by employing quantitative measures.

## Data Availability

The data are available upon request to the authors.
